# Inversion utérine non puerpérale chronique: à propos d’un cas

**DOI:** 10.11604/pamj.2018.31.231.16668

**Published:** 2018-12-13

**Authors:** Ahmed Touimi Benjelloun, Assia Makayssi, Simohamed Ennachit, Mohamed Elkarroumi

**Affiliations:** 1 Centre Hospitalier Universitaire Ibn Rochd, Faculté de Médecine et Pharmacie, Université Hassan II de Casablanca, Casablanca, Maroc

**Keywords:** Inversion utérine, non puerpéral, hystérectomie, Uterine inversion, non-puerperal, hysterectomy

## Abstract

L'inversion utérine non puerpérale chronique est une situation clinique extrêmement rare, 85% des inversions sont puerpérales exposant l'accouchée au risque d'hémorragie de la délivrance cataclysmique. Nous rapportons le cas d'une patiente de 70 ans qui s'est présentée en consultation pour prolapsus uro-génital du 3^ème^ degré, une hystérectomie totale sans conservation annexielle a été réalisée par un double abord: voie vaginale complétée par une laparotomie mettant en évidence une inversion utérine à contenu annexielle bilatérale. La voie abdominale a permis en plus d'une bonne exposition chirurgicale, l'exclusion d'un contenu digestif ou urinaire pris dans l'inversion avant la réalisation de l'hystérectomie. Bien que rare et de diagnostic difficile, l'inversion utérine non puerpérale aiguë est une urgence médico-chirurgicale.

## Abstract (en)

Chronic non puerperal uterine inversion is an extremely rare clinical condition. Puerperal inversion occurs in 85% of cases exposing women to the risk of postpartum cataclysmic hemorrhage. We here report the case of a 70-year old patient presenting with urogenital prolapse Stage III. Total double-approach hysterectomy without adnexal conservation was performed: vaginal approach complemented by laparotomy showing uterine inversion with bilateral adnexa pulled into the inversion. Abdominal approach allowed good surgical exposure, exclusion of gastrointestinal or urinary contents drawn into the inversion before hysterectomy. Although rare and difficult to diagnose, acute non-puerperal uterine inversion is a medical-surgical emergency.

## Introduction

L'inversion utérine se définit comme étant le retournement de l'utérus en doigt de gant ou « invagination utérine ». C'est une pathologie rare, plus fréquente en obstétrique qu'en gynécologie. L'inversion utérine est grave, pouvant mettre en jeu le pronostic vital du fait du choc hypovolémique qu'elle entraîne [1]. Plusieurs degrés sont décrits en fonction de la localisation du fond utérin: 1^er^ degré: dépression du fond vaginal en « cul de fiole »; 2^ème^ degré: franchissement de l'orifice externe du col; 3^ème^ degré: fond utérin intra-vaginal voire extériorisé à la vulve; 4^ème^ degré: participation des parois vaginales au retournement. Cette complication peu habituelle peut donner lieu à un retard diagnostique. Le principal diagnostic différentiel alors évoqué est un fibrome accouché par le col. L'objectif de notre travail est de rapporter le cas d'une inversion utérine chronique non puerpérale associée à un prolapsus génital 3^ème^ degré.

## Patient et observation

**Figure 1:**
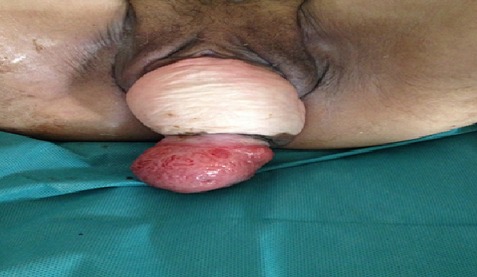
Prolapsus urogénitale 3ème degré des 3 étages irréductible, associée à une masse rouge violacée de 7cm extériorisée à la vulve

Mme J.F, âgée de 70 ans, ménopausée depuis 20 ans, 5 accouchements par voie basse sans incident notable, ayant comme antécédents notables un diabète sous antidiabétiques oraux depuis 1 an qui se présente en consultation pour gêne vaginale depuis une année associée à une dysurie, pollakiurie, brûlures mictionnelles, sans notion d'incontinence urinaire d'aggravation progressive compliquée depuis une semaine par l'extériorisation d'une masse par le vagin, sans métrorragies. L'examen clinique retrouve une vulve béante avec mauvaise trophicité cutanéomuqueuse, un méat urinaire béant sans incontinence urinaire à l'effort ni au repos. Prolapsus urogénitale 3^ème^ degré des 3 étages irréductibles, associée à une masse rouge violacée de 7cm extériorisée à la vulve (Figure 1). On note également une pâleur cutanéo-muqueuse marquée. L'échographie pelvienne par voie abdominale montre un pelvis dépourvu d'utérus. Les ovaires ne sont pas visualisés, avec importante urétérohydronéphrose bilatérale. Devant la suspicion d'inversion utérine aiguë du 3^ème^ degré, associée au prolapsus génital, nous avons opté pour une prise en charge chirurgicale avec un double abord: voie vaginale avec laparotomie. L'exploration chirurgicale a permis de confirmer le diagnostic par l'absence d'utérus dans la cavité pelvienne accompagné d'une traction vers le bas des structures normalement latéro-utérines trompes et ovaires qui apparaissent comme aspirés par le vagin.

L'intervention a débuté par voie basse en essayant de libérer et réintroduire le fond utérin après éloignement de la vessie. Une hystérectomie subtotale est réalisée par voie vaginale à distance de la vessie. La totalisation de l'hystérectomie et l'annexectomie bilatéral sont achevées par voie haute après réintroduction du col dans la cavité pelvienne et dissection vésicale. La pièce opératoire est extraite par le vagin. La tranche vaginale est suturée par du fil synthétique résorbable de polyglactin (vicryl). Puis fixée au promontoire par du fil non résorbable. Les suites opératoires ont été simples autorisant la sortie de la patiente à J3 de l'intervention. L'examen anatomo-pathologique confirme une inversion utérine sans cause évidente avec absence de malignité.

## Discussion

L'inversion utérine est une complication extrêmement rare. Au cours du post-partum, sa fréquence est estimée à 1/100 000 accouchements en France [1]. En dehors de la période puerpérale, il n'existe aucune donnée épidémiologique. Il s'agit de cas sporadiques. Cinquante-six cas ont été recensés dans la littérature entre 1976 et 2014. Dans la majorité des cas, cela concernait des femmes ménopausées ou de plus de 45 ans [2]. Quatre cas d'inversion utérine sur rhabdomyosarcome embryonnaire chez l'adolescente ont été décrits [3-5]. Deux conditions sont nécessaires pour la constitution d'une inversion utérine: une hypotonie utérine et une dilatation cervicale suffisante. Plusieurs facteurs sont impliqués dans la physiopathologie de l'inversion utérine non puerpérale: la présence d'une tumeur utérine localisée préférentiellement sur le fond utérin; sur un mur utérin fin; avec un petit pédicule tumoral; une croissance tumorale rapide; et une dilatation cervicale par distension de la cavité utérine.

L'étiologie retrouvée dans 70 à 85% des cas selon les auteurs, est le myome sous-muqueux [2]. Dans 15 à 30% des cas, il s'agit de pathologies tumorales malignes au premier rang desquelles se trouvent les sarcomes utérins (léiomyosarcome, rhabdomyosarcome embryonnaire, sarcome du stroma endométrial). Il existe deux types d'inversion utérine: puerpérale et non puerpérale ou gynécologique. Selon la gravité, on distingue quatre degrés [2]: premier degré: le fond utérin est déprimé en « cul de fiole » ou en cupule; deuxième degré: l'utérus retourné franchit l'orifice externe du col; troisième degré: le corps utérin devient intra vaginal et peut s'extérioriser complètement; quatrième degré ou inversion totale: les parois vaginales participent au retournement. Plusieurs prises en charge ont été décrites dans la littérature: un traitement conservateur quand la réduction de l'inversion utérine est possible, principalement en cas d'inversion utérine du 1^er^ ou du 2^ème^degré. Le traitement radical est privilégié en l'absence de désir de grossesse, et quasiment indispensable en cas d'inversion utérine du 3^ème^ et 4^ème^ degré [6].

L'hystérectomie peut alors être réalisée par voie vaginale, exposant l'opérateur aux difficultés techniques dues aux modifications des repères anatomiques habituels, notamment vis-à-vis des voies urinaires excrétrices (uretères et vessie). La voie abdominale est également décrite mais elle impose la réduction de l'inversion avec restitution de l'utérus dans la cavité pelvienne. L'association cœlioscopie-voie vaginale déjà décrite par l'équipe d'Auber *et al*. [7] semble être une bonne alternative permettant de confirmer le diagnostic, d'évaluer le degré d'ischémie des annexes et du vagin, et de dévasculariser l'utérus par cœlioscopie en contrôlant le pédicule utérin dès son origine. Dans la littérature, l'embolisation des artères utérines trouve son indication dans les inversions utérines non puerpérales chroniques, généralement du 2^ème^ et du 3^ème^ degré, et dans les inversions puerpérales aiguës réductibles dans le cadre d'un traitement conservateur.

## Conclusion

L'inversion utérine non puerpérale aiguë est une complication rare dont le diagnostic notamment étiologique est difficile en préopératoire devant l'urgence de la situation dans la majorité des cas. L'hystérectomie totale par double abord coelioscopique et vaginal est une technique opératoire fiable et sécuritaire.

## Conflits d’intérêts

Les auteurs ne déclarent aucun conflit d'intérêts.
